# Improved Practical Vulnerability Analysis of Mouse Data According to Offensive Security based on Machine Learning in Image-Based User Authentication

**DOI:** 10.3390/e22030355

**Published:** 2020-03-18

**Authors:** Kyungroul Lee, Sun-Young Lee

**Affiliations:** 1 R&BD Center for Security and Safety Industries (SSI), Soonchunhyang University, Asan-si, Chungnam 31538, Korea; carpedm@sch.ac.kr; 2 Department of Information Security Engineering, Soonchunhyang University, Asan-si, Chungnam 31538, Korea

**Keywords:** practical security, offensive security, user authentication, machine learning, vulnerability analysis

## Abstract

The objective of this study was to verify the feasibility of mouse data exposure by deriving features to improve the accuracy of a mouse data attack technique using machine learning models. To improve the accuracy, the feature appearing between the mouse coordinates input from the user was analyzed, which is defined as a feature for machine learning models to derive a method of improving the accuracy. As a result, we found a feature where the distance between the coordinates is concentrated in a specific range. We verified that the mouse data is apt to being stolen more accurately when the distance is used as a feature. An accuracy of over 99% was achieved, which means that the proposed method almost completely classifies the mouse data input from the user and the mouse data generated by the defender.

## 1. Introduction

Due to the emergence of an online society, the need for technologies for online user authentication has been on the rise. A representative online user authentication technology is a password-based authentication technology where the information, ID, and password that necessary for authentication is input from the keyboard [1]. However, attackers have come up with keyboard data attack techniques that they use to expose keyboard data input from a user [2,3,4]. To counteract these security threats of keyboard data, researchers have come up with an image-based authentication technology [5,6,7]. This technology uses a specific location chosen by the user as a password in the image displayed on the screen. Since in this image-based authentication, the password is input from the mouse, security threats of the password-based user authentication methods that result from the exposure of the keyboard data are avoided. Nevertheless, just like with the keyboard data, a security threat has been found that does not ensure the security of image-based authentication because of the exposure of mouse data [8,9,10,11]. 

Specifically, in image-based authentication, the authentication information that must be protected is image data to be displayed and mouse-click data to be input. In this study, we focused on mouse-click data. The operating system manages the mouse position for the interaction with the user and provides API, GetCursorPos() function, to obtain the current mouse position on the screen. Thus, an attacker can call the API periodically at short intervals to track the movement of the mouse data that is input from the user, i.e., the attacker can steal the mouse position that the user inputs, which means that image-based authentication is neutralized by exposing the password. To counteract this vulnerability, the mouse data protection technology has emerged. The key idea of this technology is not to prevent the exposure of mouse data, but to confuse the attacker so that the actual mouse position input from the user is unknown. Namely, by using the SerCursorPos() function to randomly generate the mouse position known only to the defender, the attacker cannot differentiate the coordinates input from the mouse from the coordinates generated by the defense tool [10]. As a result, the attacker collects both the random mouse data and the real data input by the user, which means that the attacker does not steal the user’s password in the image-based authentication, because the attacker does not know the random location generated by the defender. 

Accordingly, attackers have come up with attack techniques to track mouse movements by neutralizing the mouse data protection technique. Among them, an attack technique based on machine learning that effectively classifies the actual mouse position input by the user among all collected mouse data positions has emerged [12]. This technique not only requires the attacker to have a high level of attack techniques, but has also a high accuracy rate. Specifically, the maximum accuracy is 98%, and in order to classify the mouse position with high accuracy, the feature of the datasets for machine learning models is composed of the collectable X and Y coordinates as well as the elapsed time of the collected mouse data. Although this technique has a high accuracy demand, attackers still require a technique to completely steal mouse data. Therefore, in this study, we examined the feasibility of an attack technique that improves the accuracy by constructing features to the existing datasets. 

The contributions of this paper are as follows:

- Mouse data attack technique using the existing machine learning has defined the elapsed time and collected coordinates as features and has a high attack success rate. In this study, we improved the accuracy of the attack success rate by constructing the distance of the collected coordinates to the features. Hence, attackers can more effectively steal passwords by tracking the mouse movement input from the users in image-based authentication;
- The accuracy of the existing attack technique is 98%; that is, attackers have a 98% attack success rate. Although this attack has a high accuracy, 2 of 100 do not track the mouse position. Moreover, when the user moves the mouse once, attackers may be unable to track the mouse movement almost completely, considering that many mouse coordinates are generated quickly. On the other hand, the attack technique proposed in this study has more than 99% accuracy of many machine learning models in multiple datasets, which means that the attack success rate is higher than that of the existing attack techniques. Overall, the proposed attack technique tracks the mouse movement input by the user more effectively in image-based authentication and can steal passwords with high probability.

This paper is organized as follows: Section 2 describes the mouse data transfer process and the existing mouse data attack and defense techniques that are necessary for understanding the proposed mouse data attack technique. Section 3 presents the dataset configuration and features for the proposed attack technique, and Section 4 shows the experiment results. Finally, we conclude this paper in Section 5.

## 2. Related Studies

This section describes the process of transferring mouse data from a mouse device to an application program, which is part of the important information in the image-based authentication technology.

### 2.1. Mouse Data Transfer Process

**Figure 1:**
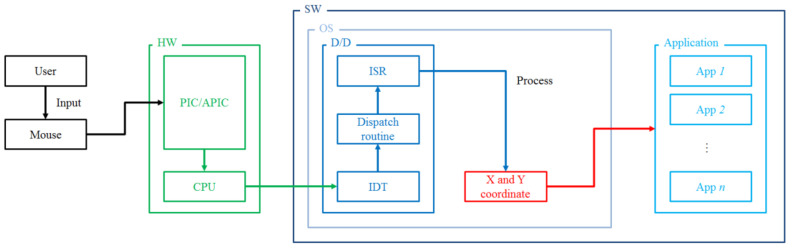
Mouse data transfer process.

The operating system supports the interaction between the mouse device and the user and manages the cursor position on the screen based on the data transmitted from the mouse device. Data transferred from the mouse device is collected by a device driver for the mouse device of the operating system, and the collected mouse data are transferred to the application program to support the interaction with the user. Figure 1 shows the mouse data transfer process.

The host PC uses interrupts to handle requests for input and output from the devices, i.e., when the user moves the mouse device, the mouse device generates an interrupt to the host PC (CPU), which calls the interrupt handler in the device driver of the operating system to handle it, and the mouse data is processed in that handler and passed to the application program.

### 2.2. Mouse Data Attack and Defense Techniques

The operating system provides a mouse cursor for interacting with the user and positions the mouse cursor based on the mouse data input by the user. For the interaction to occur, the operating system must manage the position of the mouse cursor and provide APIs related to the mouse, GetCursorPos() function, for supporting requests from application programs. Therefore, the attacker collects the mouse position on the screen, which represents the movement of the mouse input by the user, by periodically calling this GetCursorPos() function at short intervals. 

To protect the mouse movement from being exposed by the attack technique, a defender has been proposed to prevent stealing the mouse data input from the actual user rather than preempting the mouse data [10]. This technique prevents exposure of the actual mouse data by generating random mouse positions in a short period of random times by the defender. Attackers collect both the random mouse positions generated by the defender and the actual mouse positions input by the user, however, they cannot classify the mouse positions since the random mouse positions generated by the defender are not known. Therefore, the defender effectively prevents mouse data from being exposed. 

An attacker requires a technology to completely steal mouse data, and the feasibility of data stealing has been studied when a mouse data protection technology is applied [12]. In this study, both random mouse data generated by the defender and actual mouse data input by the user were collected, and the security of mouse data was verified by classifying mouse data using machine learning models by configuring datasets from the collected data. As a result, this technique verified that the mouse data was stolen with a high attack success rate of 98%, by applying the machine learning.

In this study, we proposed a method to improve the accuracy by the defining and analyzing the feature appearing between mouse coordinates input by the user.

## 3. Feature Extraction and Dataset Configuration 

### 3.1. Feature Extraction

**Figure 2:**
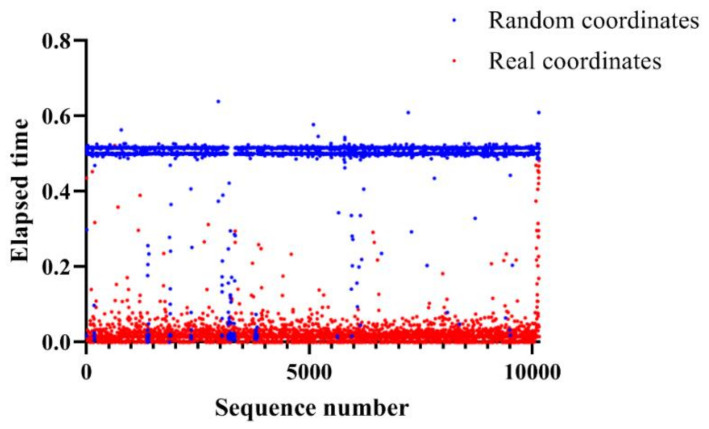
Distribution of the coordinates according to the elapsed time.

The proposed method uses an existing attack technique that collects all mouse positions by periodically calling the GetCursorPos() function. As described in Section 2.2, simply using this attack technique does not guarantee mouse data attack success. However, in order to effectively steal mouse data, we analyze the characteristics of mouse coordinates and configure features based on the analyzed results. The previous attack technique based on machine learning used the elapsed time and the collected coordinates as a feature. To analyze the characteristics of this feature, we derived the distribution of coordinates with respect to elapsed time, X coordinates, Y coordinates, distance between X coordinates, and distance between Y coordinates. Figure 2 shows the distribution of the coordinates according to the elapsed time.

**Figure 3:**
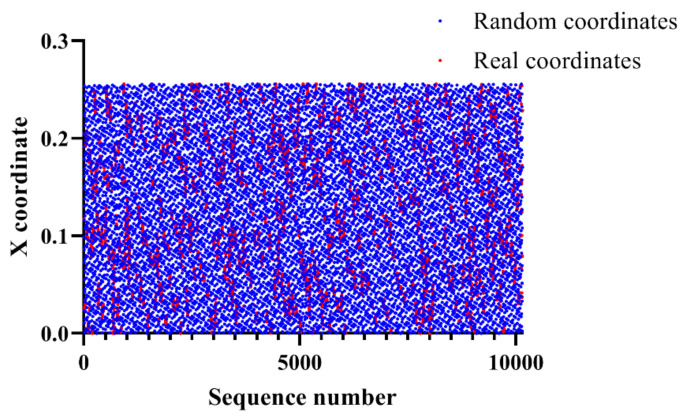
Distribution of the coordinates according to the X coordinate.

In Figure 2, all points are a distribution of the coordinates according to the elapsed time. The red dots are the real coordinates while the blue dots are the random coordinates. The distribution of random coordinates is relatively periodic and has a distribution close to 0.5, because random coordinates are generated by the defense tool. Conversely, the distribution of real coordinates is aperiodic and has a distribution near zero, because real coordinates are input from the user. Therefore, we hypothesized that it is possible to classify random coordinates and real coordinates based on the elapsed time. Figure 3 shows the distribution of the coordinates with respect to the X coordinate.

**Figure 4:**
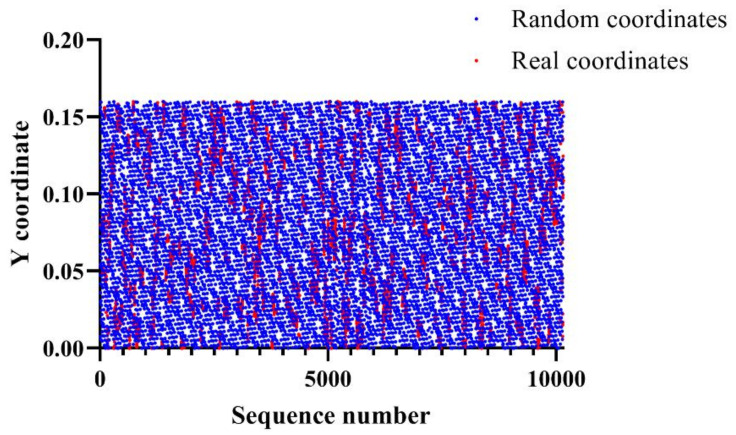
Distribution of the coordinates according to the Y coordinate.

Specifically, all points are a distribution of the coordinates according to the X coordinate. The red dots are the real coordinates while the blue dots are the random coordinates. Random coordinates have an overall wide distribution ranging from 0 to 0.24, while real coordinates have an intensive distribution with a relatively arbitrary range. Therefore, we hypothesized that it is possible to classify random coordinates and real coordinates based on X coordinates. Figure 4 shows the distribution of the coordinates according to the Y coordinate.

**Figure 5:**
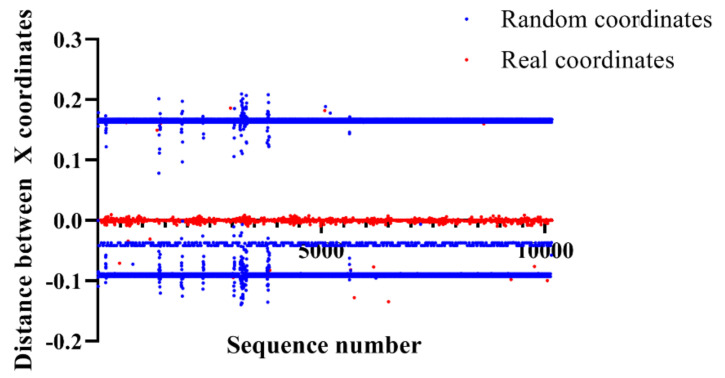
Distribution of the coordinates according to the distance between previous X coordinates and current X coordinates.

All points are a distribution of the coordinates with respect to the Y coordinate. The red dots are the real coordinates while the blue dots are the random coordinates Random coordinates have an overall wide distribution ranging from 0 to 0.16, while real coordinates have an intensive distribution with a relatively arbitrary range. Therefore, we hypothesized that it is possible to classify random coordinates and real coordinates based on Y coordinates. Figure 5 shows the distribution of the coordinates with respect to the distance between previous X coordinates and current X coordinates.

**Figure 6:**
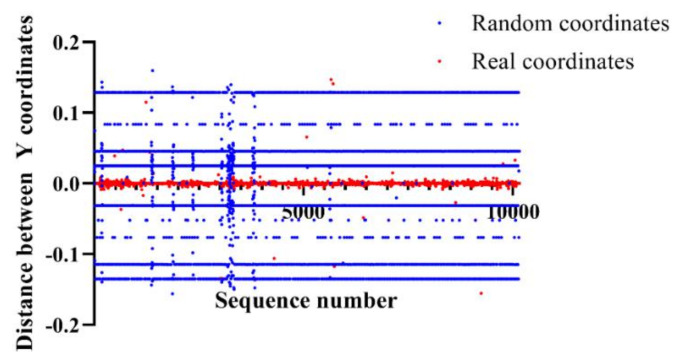
Distribution of the coordinates according to the distance between previous Y coordinates and current Y coordinates.

All points are a distribution of the coordinates with respect to the distance between previous X coordinates and current X coordinates. The red dots are the real coordinates while the blue dots are the random coordinates. Random coordinates have intensive distributions at 0.16, −0.04, and −0.08, while real coordinates have an intensive distribution range from −0.01 to 0.01. Therefore, we hypothesized that it is possible to classify random coordinates and real coordinates based on the distance between previous X coordinates and current X coordinates. Figure 6 shows the distribution of the coordinates with respect to the distance between previous Y coordinates and current Y coordinates.

All points are a distribution of the coordinates with respect to the distance between previous Y coordinates and current Y coordinates. The red dots are the real coordinates while the blue dots are the random coordinates. Random coordinates have intensive distributions at 0.12, 0.04, 0.02, −0.03, −0.11, and −0.13, while real coordinates have an intensive distribution range from –0.01 to 0.01. Therefore, we hypothesized that it is possible to classify random coordinates and real coordinates based on the distance between previous Y coordinates and current Y coordinates. We analyzed the distribution of coordinates based on the elapsed time, X coordinates, Y coordinates, distance between X coordinates, and distance between Y coordinates. After a comprehensive analysis, we derived that each distribution has a distribution that classifies random and real coordinates. Consequently, defining these characteristics as features is expected to classify mouse data more precisely. 

To classify the actual mouse data input by the mouse, the mouse data collected from the attack tool is composed as a dataset, with data collected by setting four periods, 50 ms, 100 ms, 250 ms, and 500 ms. As in a previous study [12], datasets collected using the existing attack technique were used in the same way, and the distance between the previous coordinates and the current coordinates was added as a feature to configure datasets for the experiment. Specifically, the collected X and Y coordinates, the elapsed time, and the distance between the previous coordinates and the current coordinates are defined as features.

### 3.2. Feature Definition

In this subsection, we describe the features defined in this paper. The previous research defined time difference and coordinates as features, while this paper defined five features for machine learning models. The defined features are the elapsed time, X coordinates, Y coordinates, distance between X coordinates, and distance between Y coordinates. 

#### 3.2.1. Elapsed Time

Elapsed time is the time when the X and Y coordinates are acquired in ns unit. We defined the difference between the time when the previous mouse data was collected and the time when the current mouse data was collected as a feature as shown in Equation (1), and used it as the elapsed time feature.

elapsed time = *T_C_ – T_P_*. (1)

#### 3.2.2. X Coordinates and Y Coordinates

X coordinate features and Y coordinate features are the X and Y coordinates data of all the random and real coordinates, respectively. Each coordinate is represented as the X and Y coordinate on the screen, so it has a range equal to the screen size. Therefore, we preprocess these values to range from 0 to 1 in order to facilitate learning of data for machine learning models. The real mouse data input from the user collected arbitrary coordinates by manually moving the mouse device by human hand, while the random mouse data generated by the defense tool generated random coordinates obtained by calling a random function. Specifically, random coordinates are generated periodically by a timer function called at 50 ms, 100 ms, 250 ms, and 500 ms intervals. 

#### 3.2.3. Distance between X Coordinates and Distance between Y Coordinates

Distance between the X coordinates feature and distance between the Y coordinates feature are the distance from the previously collected X and Y coordinates to the currently collected X and Y coordinates. To measure the distance, the previous coordinate value is subtracted from the current coordinate value, and the final distance value ranges from 0 to 1, because the coordinates have values between 0 and 1 after preprocessing. Nevertheless, if the value is subtracted, it is expressed as a negative number, therefore, to facilitate the data learning for machine learning models, the absolute value is obtained to have a positive value as shown in Equations (2) and (3). These distances are used as features because it is easy to classify the data as the coordinates have sequential characteristics when the user moves the mouse device.

Distance between X coordinates = | C_Xcor_ – P_Xcor_ |, (2)

Distance between Y coordinates = | C_Ycor_ – P_Ycor_ |. (3)

### 3.3. Machine Learning Models and Performance Evaluation

The machine learning models utilized in this study are KNN [13], Logistic Regression [14], Decision Tree [15], Random Forest [16], Gradient Boosting Regression Tree [16], Support Vector Machine (SVM) [17], and MLP [18]. KNN classifies data based on the decision boundary, and Logistic Regression classifies data using linear function as shown in Equation (4). Decision Tree learns data by repeating yes and no questions until it reaches a decision, and SVM classifies the data based on the distance from the data points located at the decision boundary, as shown in Equation (5). Finally, MLP classifies data through deep learning by configuring hidden units.

(4) $$\hat{y}=w[0]\times x\left[0\right]+w[1]\times x\left[1\right]+\ldots +w[p]\times x\left[p\right]+b>0,$$

(5) $$k_{rbf}(x_{1},\;x_{2})=\exp(-\gamma \;||\;x_{1}-x_{2}\;||\;2).$$

To evaluate the performance of machine learning models, we used accuracy, precision, recall, and F1-score for each model. Accuracy refers to an attack success rate as shown in Equation (6), and precision refers to the ratio of the results that are actually true to the total results as shown in Equation (7). Recall is the ratio of the results classified by the model as true to the total result as shown in Equation (8), and the F1-score is the harmonic mean of precision and recall as shown in Equation (9).

(6) $$\mathrm{ACCURACY}=\frac{TP+TN}{TP+TN+FP+FN\;}\;$$

(7) $$\mathrm{PRECISION}=\frac{TP}{TP+FP}$$

(8) $$\mathrm{RECALL}=\frac{TP}{TP+FN}$$

(9) $$F1-\mathrm{score}=2\times \frac{PRECISIONࢫRECALL}{PRECISION+RECALL}$$

## 4. Experiment Results

This section describes the experiment results of model validation, accuracy, precision, recall, F1-score, and AUC to evaluate the performance of the proposed attack technique in order to prove its contribution. The machine learning models used in this paper are KNN, Logistic Regression, Decision Tree, Random Forest, Gradient Boosting Regression Tree, SVM, and MLP. For the experiments, the training sets, validation sets, and test sets were classified into any number to overcome overfitting and underfitting. Table 1 shows the results of each set based on dataset 1-1. For learning, we divided the training set and the test set into a ratio of 3:1 for all the data, and for the verification, the training set divided the training set and the validation set into a ratio of 3:1. Moreover, we set the seed values to include data randomly in each set when dividing the data for reasonable learning.

**Figure 7:**
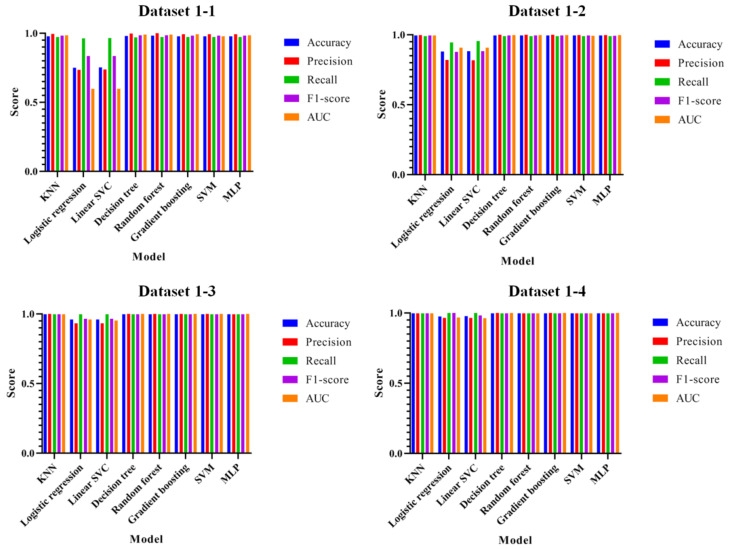
Performance evaluations of accuracy, precision, recall, F1-score, and AUC for datasets 1-1 to 1-4.

As shown in the table, the training set has the highest score with a Random Forest of 1.0, and the validation set and the test set have a score of 0.98 for most of the models except for Logistic Regression. To evaluate the performance of the proposed attack technique, Figure 7 shows the evaluations of accuracy, precision, recall, F1-score, and AUC for datasets 1-1 to 1-4.

As shown in the figure, most of the datasets work well except for the Logistic Regression. The lowest performances are 0.75, 0.88, 0.96, and 0.97 for the Logistic Regression in datasets 1-1, 1-2, 1-3, and 1-4, respectively. The highest performances are 0.981 for the Random Forest in dataset 1-1, 0.995 for the Decision Tree, Random forest, and Gradient Boosting Regression Tree in dataset 1-2, 0.998 for the KNN, Decision Tree, Random Forest, Gradient Boosting Regression Tree, and SVM in dataset 1-3, and 0.998 for KNN, Decision Tree, Gradient Boosting Regression Tree, and SVM in dataset 1-4. Comparing between the proposed method and the existing method, the performances are improved in most of the models except for Logistic Regression and Decision Tree in dataset 1-1, and performances are improved in most of the models except for Logistic Regression in dataset 1-2. In dataset 1-3, Random Forest and SVM improved performance, while only Random Forest improved performance in dataset 1-4. Experiment results do not show an improved performance in many models. However, in most recall and AUC results, there were performance degradations of only 0.0018 on average (17 items, a total decrease of 0.031), while all other items increased by an average of 0.073 (141 items, a total increase of 10.351); hence, the increase rate is 4.055% higher than the decrease rate. 

**Figure 8:**
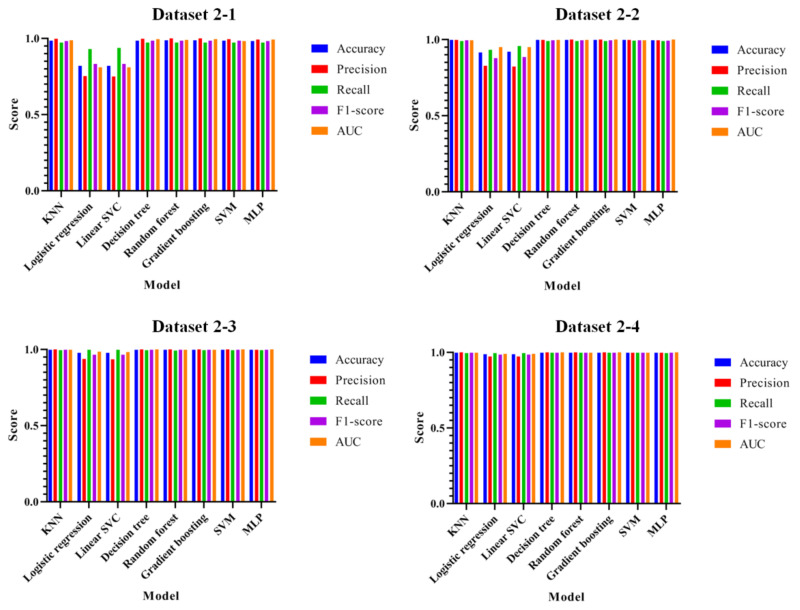
Performance evaluations of accuracy, precision, recall, F1-score, and AUC for datasets 2-1 to 2-4.

To evaluate the performance of the proposed attack technique, Figure 8 shows the results of each set of accuracy, precision, recall, F1-score, and AUC based on datasets 2-1 to 2-4.

As shown in the figure, most of the datasets show a high performance except for Logistic Regression. The lowest performances were 0.82, 0.91, 0.97, and 0.98 for Logistic Regression in datasets 2-1, 2-2, 2-4, and 2-4, respectively. The highest performances were 0.986 for the Random Forest and Gradient Boosting Regression Tree in dataset 2-1, 0.997 for the Random Forest in dataset 2-2, 0.998 for the KNN, Decision Tree, Random Forest, Gradient Boosting Regression Tree, SVM, and MLP in dataset 2-3, and 0.998 for the Decision Tree, Random Forest, Gradient Boosting Regression Tree, and SVM in dataset 2-4. Comparing between the proposed method and the existing method, performances are improved in all models in dataset 2-1 and in most of the models except for Logistic Regression in dataset 2-2. In dataset 2-3, performances are improved in Logistic Regression, Random Forest, and Gradient Boosting Regression Tree, while only Random Forest and MLP have an improved performance in dataset 2-4. Just as in datasets 1-1 to 1-4, the results do not seem to improve the performance in many models, however, most result in only a 0.001 performance degradation on recall and AUC (13 items, a total decrease of 0.129) and all other items were increased by an average of 0.054 (147 items, a total increase of 8.015); hence, the increase rate was 540%, which is higher than the decrease rate. 

**Figure 9:**
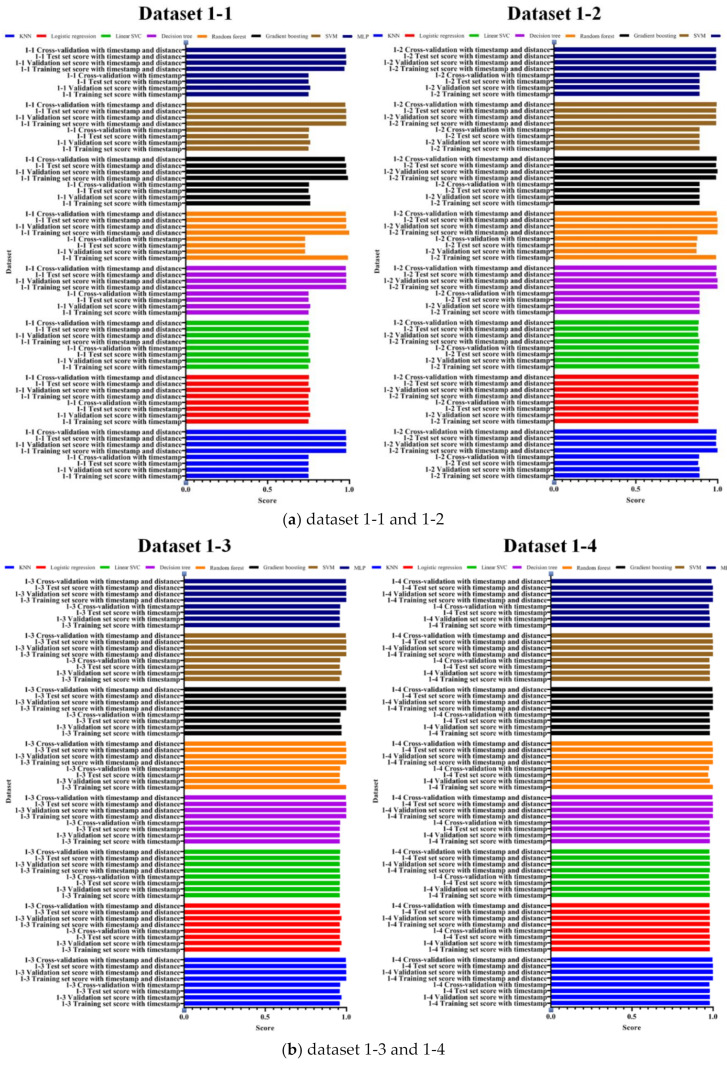

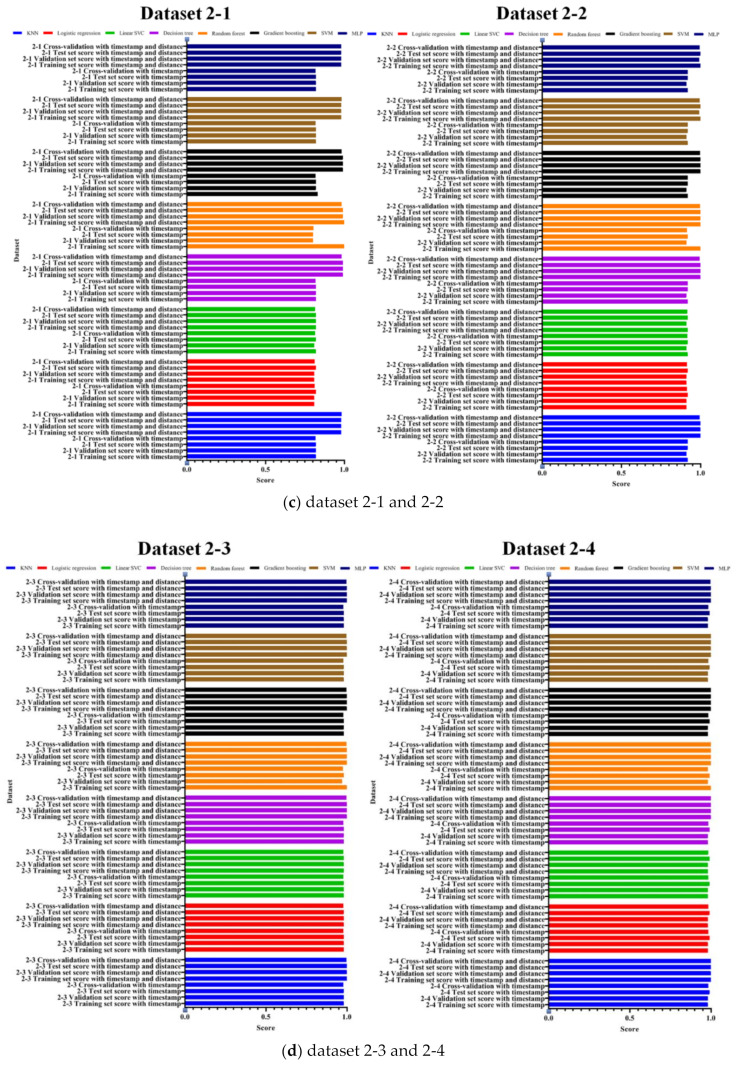
Performance evaluation of the proposed and existing methods in the training set, validation set, and test set. (**a**) dataset 1-1 and 1-2; (**b**) dataset 1-3 and 1-4; (**c**) dataset 2-1 and 2-2; (**d**) dataset 2-3 and 2-4.

As described above, in this study, we evaluated the performance of datasets including the elapsed time and the X and Y coordinates, which is the existing attack technique, and the datasets including the distance between the previous and current coordinates. Consequently, we verified through experiments that the performance of datasets with distance is higher than that of datasets with elapsed time only. Therefore, the results of the performance evaluation are changed by defining features that can be included in the datasets, and we proved the contribution of the proposed method by comparing the performance evaluations. We compared the results of the training set, the validation set, and the test set, and also compared the results of performance, accuracy, precision, recall, F1-score, and AUC. Figure 9 shows the comparison of the training set, the validation set, and the test set.

As shown in the figures, the performances of each model are represented by colors. The top four in each model are the results of the datasets that include the distance between the previous and current coordinates proposed in this study, while the bottom four are the results of the datasets that do not include the distance and were used in the previous study. Therefore, the performance of the proposed method is improved in most datasets and machine learning models. An increase in the performance trend was observed from dataset 1 to 4, and on average, all datasets including distance outperformed all the datasets that do not include distance. Moreover, all scores in datasets 1-4 and 2-4 have a near-perfect performance of over 99%, which results in a higher performance for constructing features with datasets containing distances. Regarding the changes in the training set, the validation set, and the test set, the results of most models of datasets that do not include distance have significant changes compared to models of datasets including distance that have relatively small changes. 

**Figure 10:**
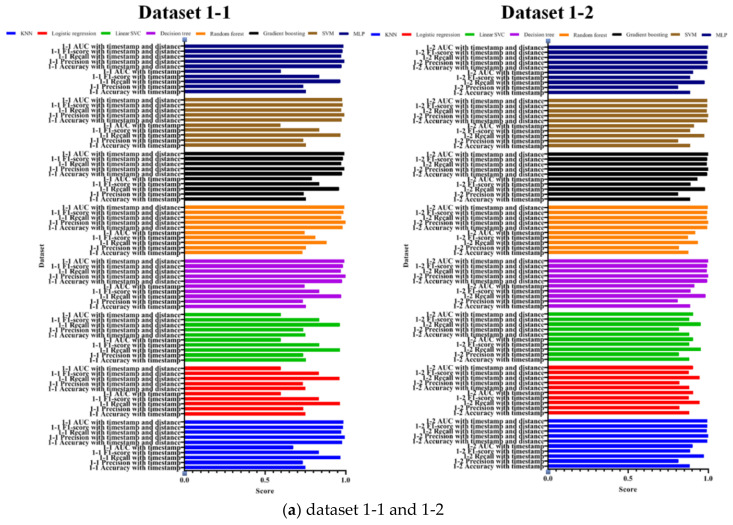

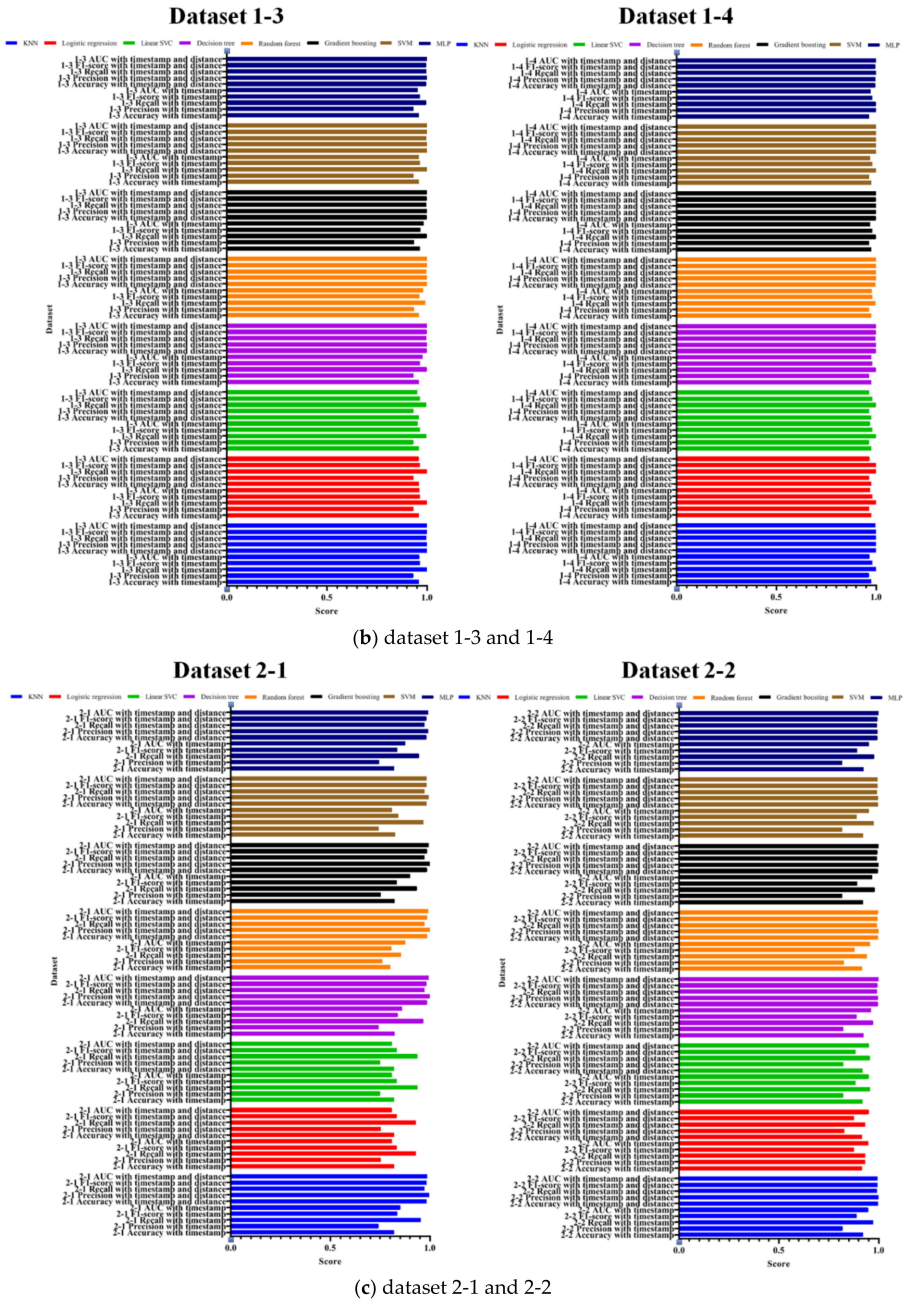

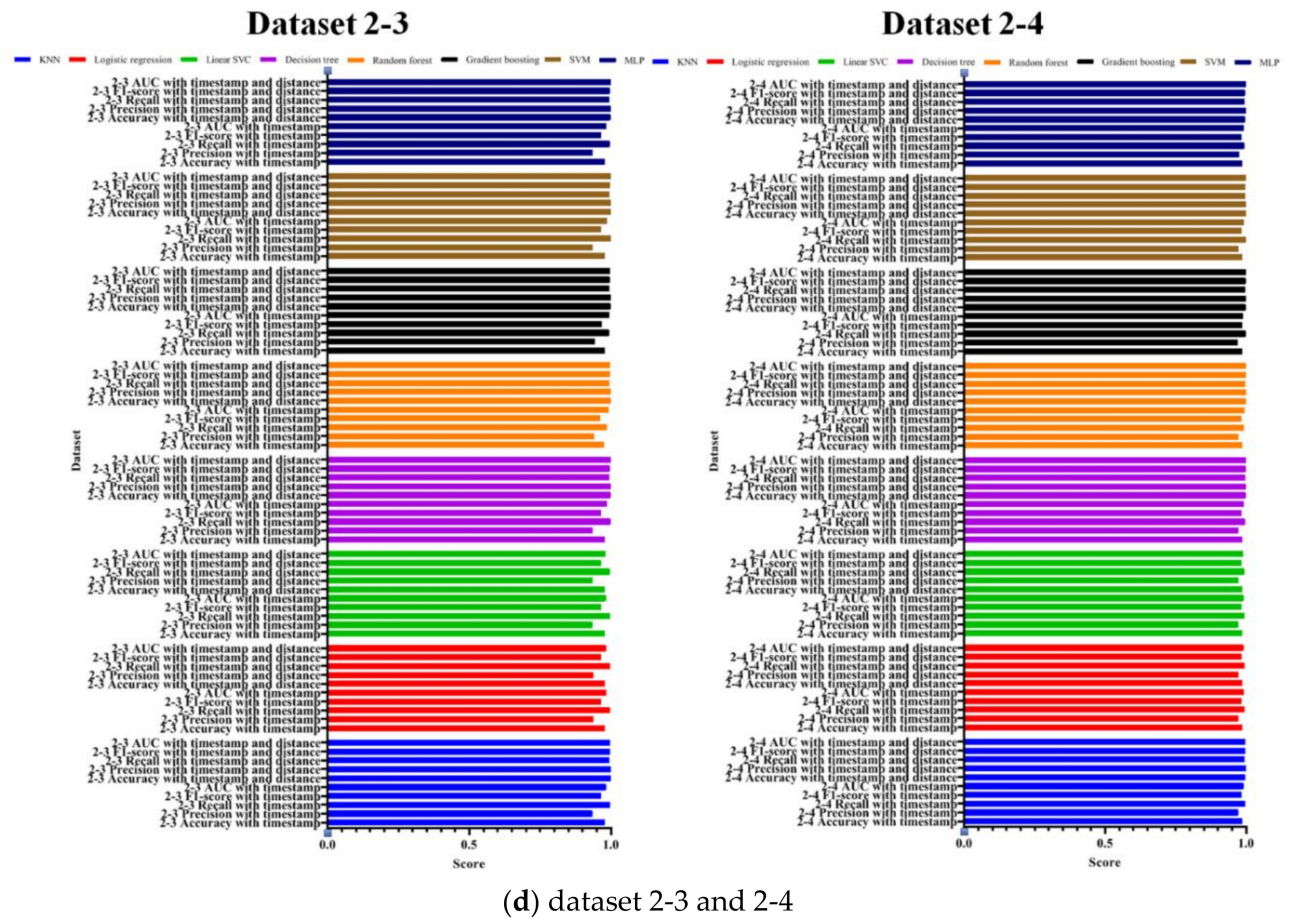
Performance evaluation of the proposed and existing methods with respect to accuracy, precision, recall, F1-score, and AUC. (**a**) dataset 1-1 and 1-2; (**b**) dataset 1-3 and 1-4; (**c**) dataset 2-1 and 2-2; (**d**) dataset 2-3 and 2-4.

As described above, the results of the training set, the validation set, and the test set showed significant differences, which means that the datasets including the distance are verified to have a high performance. Figure 10 compares the accuracy, precision, recall, F1-score, and AUC to evaluate the more practical performance.

As shown in the figures, the performances of each model are shown by colors. The top four in each model are the results of the datasets that include the distance between the previous and current coordinates as proposed in this study, while the bottom four are the results of the datasets that do not include the distance. The performance of the proposed method is improved in most datasets and machine learning models. A trend of better performance was observed as datasets went from 1 to 4, and datasets with distance have a significantly higher performance than datasets without distance. Moreover, most models of all datasets except for datasets 1-1 and 2-1 have more than 99% accuracy. In other words, most datasets have 99% accuracy, which indicates that the proposed technique effectively classifies random coordinates generated by the defender in the image-based authentication method, compared to 98%, which is the accuracy of the previous attack technique, by simply collecting mouse coordinates. Hence, the proposed method more effectively steals passwords, which are the actual mouse data input by the user.

## 5. Conclusions

This study analyzed the security of mouse data by deriving features to further improve the accuracy of existing attack techniques using machine learning based on mouse data in image-based authentication. The existing attack technique defines elapsed time and X and Y coordinates as features, classifies random mouse data generated by defender and actual mouse data input by user, with a maximum accuracy of 98%. In this study, we analyzed the distribution of coordinates based on the distance between coordinates and the elapsed time. The distance between the mouse coordinates input by the user is concentrated in a specific range. Therefore, we configured datasets that defined the distance between the previous coordinates and the current coordinates as a feature. With experiment results based on the configured datasets, we verified that the actual mouse data input by the user was classified more effectively than it was by the existing attack techniques, with an accuracy of 99%, which means that the mouse data input by the user is classified almost completely. In conclusion, the proposed attack technique can effectively steal the mouse data input by the user and the passwords as the actual mouse data input by the user.

## Figures and Tables

**Table 1:** Training set, validation set, and test set scores of dataset 1-1 with optimal parameters.

Model	Parameters	Training Set Score	Validation Set Score	Test Set Score
KNN	n_neighbors = 4	0.98	0.98	0.98
Logistic regression	C = 10, L2 regularization	0.75	0.76	0.75
Decision tree	max_depth = 6	0.98	0.98	0.98
Random forest	n_estimators = 12	1.00	0.98	0.98
Gradient boosting	learning_rate = 0.01, max_depth = 9	0.99	0.98	0.98
SVM	C = 100,000	0.98	0.98	0.98
MLP	max_iter = 1000, alpha = 0.01	0.97	0.98	0.98
